# The LIKE system, a novel protein expression toolbox for *Bacillus subtilis* based on the *liaI* promoter

**DOI:** 10.1186/1475-2859-11-143

**Published:** 2012-10-30

**Authors:** Anna A Toymentseva, Karen Schrecke, Margarita R Sharipova, Thorsten Mascher

**Affiliations:** 1 Department of Biology I, Microbiology, Ludwig-Maximilians-University Munich, Munich, Germany; 2 Department of Microbiology, Faculty of Biology and Soil, Kazan Federal University, Kazan, Russian Federation

**Keywords:** two-component system, *liaIH* operon, antibiotic-inducible promoter, cell envelope stress response, protein expression, *Bacillus subtilis*, bacitracin

## Abstract

**Background:**

*Bacillus subtilis* is a very important Gram-positive model organism of high biotechnological relevance, which is widely used as a host for the production of both secreted and cytoplasmic proteins. We developed a novel and efficient expression system, based on the *liaI* promoter (P_*liaI*_) from *B. subtilis*, which is under control of the LiaRS antibiotic-inducible two-component system. In the absence of a stimulus, this promoter is kept tightly inactive. Upon induction by cell wall antibiotics, it shows an over 100-fold increase in activity within 10 min.

**Results:**

Based on these traits of P_*liaI*_, we developed a novel LiaRS-controlled gene expression system for *B. subtilis* (the “LIKE" system). Two expression vectors, the integrative pLIKE-int and the replicative pLIKE-rep, were constructed. To enhance the performance of the P_*liaI*_-derived system, site-directed mutagenesis was employed to optimize the ribosome binding site and alter its spacing to the initiation codon used for the translational fusion. The impact of these genetic modifications on protein production yield was measured using GFP as a model protein. Moreover, a number of tailored *B. subtilis* expression strains containing different markerless chromosomal deletions of the *liaIH* region were constructed to circumvent undesired protein production, enhance the positive autoregulation of the LiaRS system and thereby increase target gene expression strength from the P_*liaI*_ promoter.

**Conclusions:**

The LIKE protein expression system is a novel protein expression system, which offers a number of advantages over existing systems. Its major advantages are (i) a tightly switched-off promoter during exponential growth in the absence of a stimulus, (ii) a concentration-dependent activation of P_*liaI*_ in the presence of suitable inducers, (iii) a very fast but transient response with a very high dynamic range of over 100-fold (up to 1,000-fold) induction, (iv) a choice from a range of well-defined, commercially available, and affordable inducers and (v) the convenient conversion of LIKE-derived inducible expression strains into strong constitutive protein production factories.

## Background

*Bacillus subtilis* is a widely exploited bacterium for basic research, but also industrial and biotechnological applications [1] owing to the ease of genetic manipulation, a systems level understanding of its genome and physiology [2-4], its efficient protein secretion systems [5], non-pathogenic GRAS-status [6] and well-characterized mechanisms for gene expression [7]. Over the years, numerous genetic devices and expression systems have been developed for this organism to facilitate the production of homologous or heterologous proteins [7-14], usually based on strong inducible promoters. Such systems can either be integrated into the chromosome or located on replicative plasmids to increase the gene copy number under the control of the inducible promoter.

A number of new expression systems based on induction by peptide antibiotics were described for Gram-positive bacteria [9,15,16]. The nisin-controlled gene expression (NICE) system was developed for different species of *Lactococcus* and *Lactobacillus* and allows the production of the desired proteins in high amounts (comparable to other expression systems), reaching a maximum 3 h after nisin induction [15,16]. A very similar subtilin-regulated expression system (SURE) was recently constructed for *B. subtilis*[9]. Both systems enable the controlled overexpression of a variety of homologous and heterologous proteins and enzymes and show a number of advantages to other control elements, such as the strict control of gene expression, no leakage of the promoter regulation under non-inducing conditions, high levels of expression upon induction and almost no limitations in the choice of sugar-containing media [9,15]. For the use in *B. subtilis*, the SURE system has several advantages over the NICE system: (i) The SURE system only requires a single plasmid, thereby ensuring a stable expression platform; (ii) the expression levels achieved by the SURE system are significantly higher; and (iii) it also requires lower concentrations of the inducer molecule [9,17].

Despite significant progress in the field, no exisiting system works equally well for all proteins and none of the existing expression systems for *B. subtilis* is without pitfalls or limitations. While the SURE system represents a major improvement, its inducer, the lantibiotic subtilin, is not commercially available. Instead, culture supernatant of the lantibiotic producer must be used, which introduces a source of variation and requires testing the potency each time a new supernatant is used for induction. Therefore, novel tightly controllable gene expression systems are still in demand to expand and complement the existing repertoire in order to find the optimal solution for a given protein to be produced in *B. subtilis*.

Here, such an addition to the existing bioengineering toolbox for *B. subtilis* will be described. The LIKE (from the German “*LIa-Kontrollierte Expression”*) system is based on the cell envelope stress-responsive *liaI* promoter. This promoter was initially identified in the course of studies on the response of *B. subtilis* to the presence of harmful concentrations of various cell wall antibiotics [18]. The underlying regulatory network of the cell envelope stress response in this organism is rather complex and consists of at least four extracytoplasmic function (ECF) σ factors and a similar number of two-component systems (TCS) and has been extensively studied [19,20]. One such TCS, LiaRS, is a central player in the envelope stress response network of *B. subtilis*. It strongly responds to antibiotics that interfere with the lipid II cycle, such as bacitracin. Activation of the LiaRS system of *B. subtilis* specifically leads to the strong induction of a single target promoter, P_*liaI*_, which drives the expression of the *liaIH* operon. This promoter is basically shut off in the absence of inducing condition during logarithmic growth and shows an impressive dynamic range of over 100- up to 1,000-fold in the presence of suitable stimuli [21-23].

Because of its specificity and sensitivity, P_*liaI*_ has already been developed as a powerful screening tool for mechanism-of-action studies of novel peptide antibiotics interfering with envelope integrity [22,24,25]. But its tightly regulated, concentration-dependent and highly dynamic behavior also makes this promoter a very promising candidate for the development of a novel gene expression system. This prospect is further supported by transcriptome studies of mutants that are constitutively switched Lia-ON or Lia-OFF, which revealed a very specific response with only very few genes being indirectly affected [23]. Moreover, *B. subtilis* is highly resistant to bacitracin, a commercially available compound, which can be used as an ideal inducer to activate P_*liaI*_-driven gene expression in growing cultures of *B. subtilis*. Moreover, a simple gene deletion can convert the inducible into a high-level constitutive promoter activity. Based on these traits of P_*liaI*_, we developed vectors and strains to apply this promoter as a powerful protein expression system in *B. subtilis*.

## Results and discussion

### Features of the native *liaI* promoter (P_*liaI*_)

**Figure 1:**
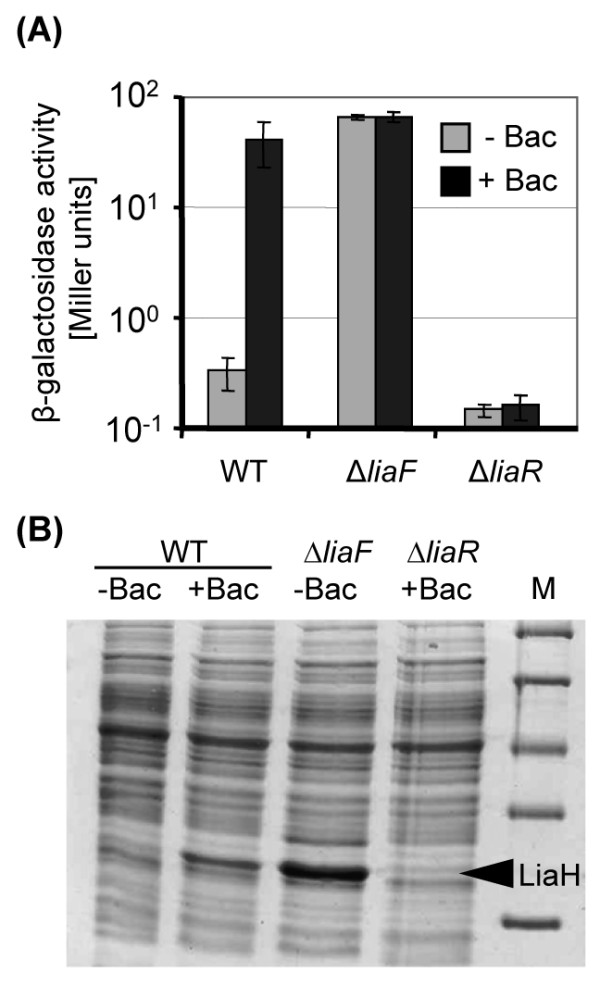
**Activity of the native *liaI *promoter (P**_***liaI***_**) as monitored by (A) promoter-reporter gene fusions and (B) SDS-PAGE.** (A) β-Galactosidase reporter assays of a P_*liaI*_*-lacZ* fusion in the wild type W168 (TMB016) and the corresponding *liaF* and *liaR* mutants (TMB331 and TMB020, respectively) in the presence and absence of bacitracin (Bac; final concentration 50 μg/ml). The assay was performed as previously described [21], the promoter activity is expressed as Miller units (β-galactosidase activity normalized against cell density). (B) SDS-PAGE of the soluble protein fraction (15 μg/lane) of the wild type (WT) and isogenic *liaF* and *liaR* mutants, challenge for 30 min with bacitracin as described above. The position of the band corresponding to the LiaH protein is marked. The identity of LiaH was verified by mass spectroscopy. M, molecular weight markers.

Previously, we have characterized the cell envelope stress-inducible promoter P_*liaI*_, which controls the expression of the *liaIH* operon in *B. subtilis*. During normal logarithmic growth, this promoter is virtually switched off and hence does not show any significant basal activity. In the presence of suitable inducers such as the cell wall antibiotic bacitracin, it strongly responds in a concentration-dependent manner, resulting in a more than 100-fold increased activity already 5–10 min after the addition of bacitracin. This activity strictly depends on the activity of the response regulator LiaR [21-23] (Figure 1A). This tight regulation and the impressive strength of P_*liaI*_ under inducing conditions are illustrated by the protein gel shown in Figure 1B, which demonstrates that even from the native P_*liaI*_, present in single copy on the chromosome, LiaH is the predominant protein produced under inducing conditions, as has already been indicated previously by 2D gelectrophoresis [23]. These features make P_*liaI*_ a very promising candidate for developing a novel protein-expression system for the Gram-positive model organism *B. subtilis*, which is widely used in the biotechnological industry as a protein production host [1]. To achieve this, the *liaI* promoter was first sequence-optimized and then integrated into two expression vectors. Moreover, a set of suitable expression strains was developed and evaluated to further improve the promoter strength while simultaneously avoiding the metabolic burden of overexpressing the native target proteins of LiaR-dependent gene regulation, LiaI and LiaH [23], as indicated in Figure 1B.

### Design and construction of P_*liaI*_-based expression vectors and *B. subtilis* protein production strains for the LIKE system

**Figure 2:**
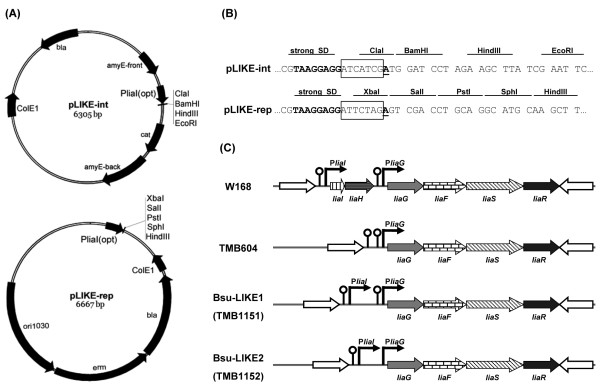
**Vectors and strains of the LIKE system.** (**A**) Vector maps of integrative plasmid pLIKE-int and *E. coli*/*B. subtilis* shuttle vector pLIKE-rep. Abbreviations: P_*liaI*(opt)_, *liaI* promoter with optimized SD sequence; *bla*, ampicilin resistance; *erm*, erythromycin resistance; *cat*, chloramphenicol resistance; ColE1, origin of replication for *E. coli*; ori1030, origin of replication for *B. subtilis*. (**B**) Sequence of the multiple cloning sites for each plasmid. The optimized SD sequence is indicated in bold, the 7 bp ‘spacer’ is boxed, the first nucleotide of the coding sequence is underlined. For pLIKE-int, ClaI can be used as restriction enzyme to create an ATG start codon. For pLIKE-rep, XbaI must be used to reconstruct the ATG start codon. (**C**) Schematic representation of the *liaI* operon and genotype of deletion strains constructed in this study. Open reading frames are depicted as solid arrows. Promoters of the genes indicated by thin arrows, terminators by hairpin symbols.

A closer inspection of the *liaI* promoter sequence revealed a poorly conserved Shine-Dalgarno sequence (SD) with a suboptimal spacing to the *liaI* start codon (data not shown). As a first step in developing a P_*liaI*_-derived bacitracin-inducible expression system, we therefore optimized its SD sequence by introducing a strong *B. subtilis* ribosome binding site (TAAGGAGG) with an optimal spacing of seven nucleotides upstream of the start codon, which was used for all subsequent constructions and will be referred to as P_*liaI*(opt)_ from now on (Figure 2). This optimized SD sequence is well established for *B. subtilis*[26,27], and provides optimal complementarity to the 3’-end of the 16S rRNA, thereby increasing the ribosome’s affinity to the mRNA and enhancing the translation initiation efficiency.

For the construction of new P_*liaI*_-derived bacitracin-inducible gene expression systems, we chose two vectors as backbones: the *E. coli*/*B. subtilis* shuttle vector pGP380, and pDG1662 for ectopic integration at the *amyE* locus of *B. subtilis*[28,29], thus enabling both expression from a multi-copy replicative vector, as well as the stable chromosomal integration at single copy. The optimized regulatory element P_*liaI*(opt)_ was amplified by PCR and cloned into the two vectors (see Materials and Methods for details) resulting in the expression vectors pLIKE-rep and pLIKE-int, respectively (Figure 2A/B).

Previous work has demonstrated that the *liaIH* operon is the only relevant target of LiaFSR-dependent gene expression, and that activation of P_*liaI*_ results in a strong accumulation of LiaH in the cytosol (Figure 1B) [23]. Based on the organization and expression of genes in the *liaIH-liaGFSR* locus, activation of P_*liaI*_ also leads to an increased expression of *liaGFSR*, due to read-through transcription [22]. Such positive autoregulatory feedback loops often have beneficial effects on the activity of their target genes [30]. Hence, it might be desirable to maintain this feedback loop. On the other hand, the observed very strong production of the native LiaFSR-target proteins LiaIH is not desired in a protein production host, since it depletes the cells of energy, amino acids and ribosomes required for heterologous protein production.

To account for these two opposing goals, we constructed a number of clean deletion mutants as potential hosts of the LIKE-system. The features of the resulting strains are summarized in Figure 2C. Strain TMB604 lacks both the *liaIH* operon including the native *liaI* promoter. Hence, no autoregulation can occur under inducing conditions. In contrast, strains TMB1151/TMB1152, which are also deleted for the *liaIH* operon, still maintain P_*liaI*_ and therefore autoregulation. They differ in the presence or absence of the weak terminator located downstream of *liaH* (Figure 2C).

As a measure for P_*liaI*(opt)_-dependent protein production in the two expression plasmids, *gfpmut1* gene was used as a reporter gene [31]. Translational fusions of P_*liaI*(opt)_ with *gfpmut1* were constructed in both pLIKE-int and pLIKE-rep and subsequently introduced into the aforementioned *B. subtilis* strains.

### Evaluation of the LIKE-system, based on the bacitracin-induced GFP production

The range of inducers for the envelope-stress responsive LiaFSR three-component system is well-defined and includes, amongst others, the cell wall antibiotic bacitracin [22,25]. As an inducer for protein production in *B. subtilis*, this compound has a number of advantages: (i) It is one of the strongest inducers for the Lia-system and is easily commercially available. (ii) *B. subtilis* is highly resistant against bacitracin, and even above inhibitory antibiotic concentrations, cellular damage occurs only very slowly [18,32]. (iii) The maximum P_*liaI*_ activity occurs well below the inhibitory concentration, thereby avoiding any damage to the producing cultures. (iv) In addition to its major inhibitory activity on cell wall biosynthesis, bacitracin also acts as a weak protease inhibitor [33], which can be viewed as a beneficial side effect of using this inducer. For all of these reasons, bacitracin will be used as the model inducer for the subsequently described evaluation of the LIKE expression system.

Nevertheless, it should be pointed out that a number of other compounds and conditions can also be considered as suitable alternative inducers, including antibiotics such as vancomycin or nisin, as well as non-antibiotic conditions such as alkaline shock [34], making the LIKE-system highly variable even in cases where bacitracin is not suitable for a given application (for example for heterologous protease production).

**Figure 3:**
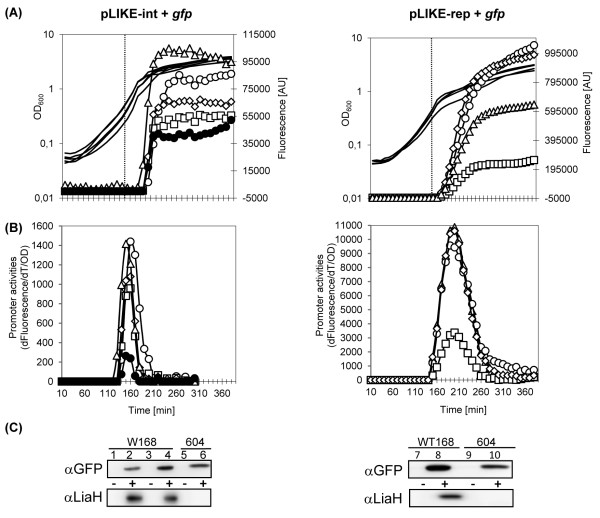
**Growth, absolute fluorescence (A) and promoter activity (B) of strains carrying translational fusion of P**_***liaI***_**-*gfpmut1 *and P**_***liaI*(opt)-**_***gfpmut1*.** Growth profiles are shown without symbols and expression by symbols: (○) (W168), (□) (TMB604), (◊) (TMB1151), (∆) (TMB1152) and (●) (TMB408). Vertical dotted line indicates time point of bacitracin addition (final conc. 30 μg mL^-1^; OD_600_~0.4-0.5). Fluorescence is expressed in arbitrary units (AU) (**C**) Western blot analysis of the cytoplasmic fractions of cells expressing the same fusions probed with LiaH or GFP antisera. Lanes 1–2, protein expression with native modified P_*liaI*_; 3–10, with optimized P_*liaI*(opt)_ in the absence (−) and presence (+) of bacitracin (final conc. 30 μg mL^-1^).

Initially, we compared the promoter activity of P_*liaI*(opt)_ between pLIKE-int and pLIKE-rep in all four different host strains described above (Figure 2C). For this purpose, the dynamics of expression of recombinant GFP was determined after bacitracin addition (30 μg mL^-1^) over the course of 4 h in growing populations. In all strains, a swift and strong accumulation of fluorescence was detected already 30 min after bacitracin induction (Figure 3A). As expected, *gfp* expression was significantly higher in strains harboring the replicative pLIKE-rep derivative (multiple copies) compared to strains with chromosomally integrated pLIKE-int derivatives (Figure 3A). In the wild type background of W168, the fluorescence intensity of the expression strain TMB1172, harboring the integrated expression plasmid, reached less than 10% of the activity measured for the otherwise identical strain TMB1176 with the replicative construct (Figure 3A).

The benefit of improving the ribosome binding site in P_*liaI*(opt)_ compared to the original SD sequence could also be demonstrated by comparing GFP production in two strains, TMB1172 and TMB408, respectively, harboring integrated expression systems. Our analysis revealed that the level of GFP expression from the wild type P_*liaI*_ promoter in strain TMB408 was significantly lower compared to P_*liaI*(opt)_-mediated expression (Figure 3A and Figure 3C, lanes 2 and 4). A deletion of the native P_*liaI*_ upstream of the *liaGFSR* operon in strain TMB604 resulted in an approx. two-fold decreased promoter activity compared to the wild type background, indicating that the presence of the autoregulatory feedback loop is important for full P_*liaI*_ activity (Figure 3A and Table 1). On the other hand, deletion of *liaH* while maintaining the native P_*liaI*_ upstream of the *liaGFSR* operon (strains TMB1151/1152) resulted in only a small increase of P_*liaI*(opt)_ activity in case of the pLIKE-int derived expression strain. This effect was more pronounced in case of the pLIKE-rep derived strains, where the promoter activity even surpassed that of the wild type (Figure 3A and Table 1). Taken together, these results demonstrate both the important role of the autoregulatory feedback and of improving the SD sequence for achieving a maximal level of GFP production.

**Table 1:** **Effect of mutations in the *liaIH *operon on the expression of translational P**_***liaI*(opt)**_**-*gfp *fusions**

**Strain**	**Relevant genotype**^**a**^		**Promoter activity (fluorescence)**^**b**^
	**Expression plasmid**	**Strain background**	
TMB408	*amyE*:: pSJ5101 (P_*liaI*_-*gfp*)	(WT168) P_*liaI*_*liaIH*_Term_^+^	264
TMB1172	*amyE*:: pLIKE-int+*gfp*	(WT168) P_*liaI*_*liaIH*_Term_^+^	1440
TMB1174		(TMB604) ∆P_*liaI*_*liaIH*	958
TMB1153		(TMB1151) ∆*liaIH*	1080
TMB1318		(TMB1152) ∆*liaIH*_Term_	1416
TMB1176	pLIKE-rep+*gfp*	(WT168) P_*liaI*_*liaIH*_Term_^+^	9570
TMB1178		(TMB604) ∆P_*liaI*_*liaIH*	3372
TMB1342		(TMB1151) ∆*liaIH*	10607
TMB1343		(TMB1152) ∆*liaIH*_Term_	10870

^a^ The terminator downstream of *liaH* is abbreviated “Term”, its presence is indicated by a “+”. ^b^ Promoter activities were calculated taking the derivative of the fluorescence divided by the OD_600_ (dGFP/dt/OD_600_) at each time point.

Determination of the P_*liaI*_ activity revealed that the window of promoter activity was narrower in case of the integrated promoter, both for activation and shut-off, relative to the replicative derivatives (Figure 3B). For the pLIKE-int derivatives, maximum promoter activity was reached already 20–30 min after addition of bacitracin and the total window of activity was less than 60 min. In contrast, pLIKE-rep derivatives required almost 60 min to reach maximum promoter activity and the total window of activity was about 120 min. But in light of the overall 10-times higher promoter activity in case of the latter, this result is maybe not too surprising.

All major conclusions drawn above were verified at the protein level by Western analysis, using antibodies against GFP and LiaH. Both proteins were not detectable in uninduced cultures, supporting the previously demonstrated tight control of P_*liaI*_ and the absence of any significant promoter activity under non-inducing conditions. Upon addition of bacitracin, both proteins accumulated to different level, depending on strain background. These studies demonstrate both the positive effect of improving the ribosome binding site and the negative effect of deleting the autoregulatory feedback loop at the level of protein production (Figure 3C).

Taken together, both pLIKE-int and pLIKE-rep were successfully established as vectors for bacitracin-dependent protein production in strains that maintain the positive autoregulatory feedback loop. While expression based on the replicative vector yields higher protein amounts, the integrative system has the advantage of genetic stability and does not require any selection.

### Effect of the inducer (bacitracin) concentration on the activity of P_*liaI*(opt)_

**Figure 4:**
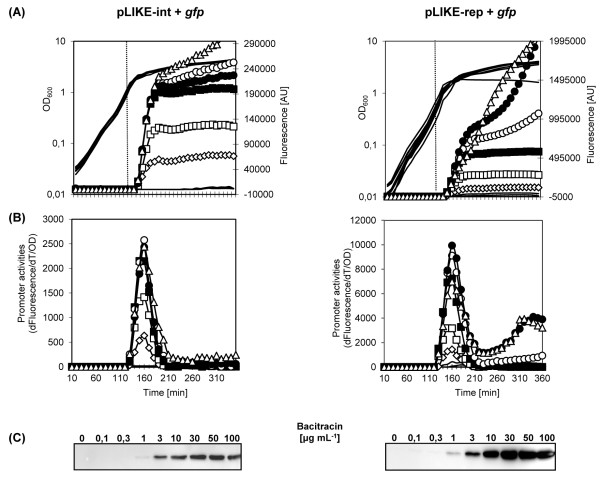
**Concentration-dependent induction of the P**_***liaI*(opt)**_**in *B. subtilis *W168 cultures.** (**A**) Growth, absolute fluorescence and (**B**) promoter activity of strains carrying translational fusion of P_*lia*I(opt)-_*gfpmut1* on plasmids pLIKE-int and pLIKE-rep treated with different concentration of bacitracin. Growth profiles are shown without symbols and expression by symbols: (◊) 1 μg mL^-1^, (□) 3 μg mL^-1^, (■) 10 μg mL^-1^, (○) 30 μg mL^-1^, (●) 50 μg mL^-1^, (∆) 100 μg mL^-1^. Vertical dotted line indicates the point of mid-log phase (OD_600_~0.4-0.5) when bacitracin was added. Fluorescence is expressed in arbitrary units (AU) (**C**) Western blot analysis revealed the amount of GFP produced by cells harboring pAT6203 (pLIKE-int) and pAT3803 (pLIKE-rep) 90 min post induction.

Next, we wanted to investigate the dynamics of P_*liaI*_ activity and the resulting GFP production as a function of the inducer concentration. It is already well established that P_*liaI*_-mediated gene expression occurs in a dosage-dependent manner, at least in case of the wild type promoter [22,23,32]. Here, we performed similar experiments, using the pLIKE-int and pLIKE-rep derivatives pAT6203 and pAT3803, respectively, in the W168 (wild type) background. The resulting strains TMB1172 and TMB1176 were inoculated in microtiter plates and challenged in the mid-exponential growth phase with increasing concentrations of bacitracin (Figure 4). The results are in very good agreement with the previous observations. The promoter activity increases as a function of the bacitracin concentration, reaching a maximum at bacitracin concentrations of about 30 μg mL^-1^ (Figure 4A/B). At higher concentrations (above 50 μg mL^-1^), the ongoing promoter activity after 250 mins indicates an ongoing bacitracin stress. Especially at the highest bacitracin concentration, 100 μg mL^-1^, the GFP yield is clearly reduced concomitant with a reduced final cell density, at least in case of the pLIKE-rep derived strain TMB1176 (Figure 4A). This result was also confirmed by Western blot analysis (Figure 4C). To ensure optimal protein production without causing severe antibiotic stress, our data suggests the use of a bacitracin concentration of no more than 30 μg mL^-1^, although this concentration may have to be optimized for individual target proteins, especially if toxicity is a problem.

### Overproduction of YdfG using the LIKE system

To demonstrate the suitability of the LIKE system for the overexpression of a heterologous protein, we performed an expression experiment using the protein YdfG of *Bacillus licheniformis*. This protein is a putative carboxymuconolactone decarboxylase. We could recently demonstrate that its gene represents the only target of the extracytoplasmic function σ factor ECF41_Bli_[35]. It consists of 148 amino acids and an estimated molecular weight of 16,6 kDa.

**Figure 5:**
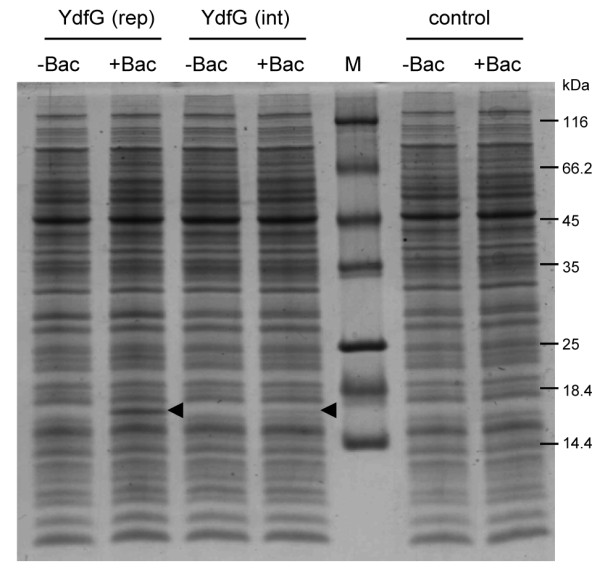
**Overproduction of YdfG using the LIKE system.** 14% tricine SDS-PAGE of total proteins (10 μg/lane) of strains TMB1566 (YdfG-rep), TMB1570 (YdfG-int), and TMB1151 (control) treated with and without 30 μg ml^-1^ bacitracin for 30 min. The position of the band corresponding to the protein YdfG is marked. M, molecular weight marker (in kDa).

Based on the results shown in Figures 3 and 4, we used strains TMB1151 and TMB1152 as expression hosts for the pLIKE-rep+His_6_-*ydfG* (pKSLIKEr01) and pLIKE-int+His_6_-*ydfG* (pKSLIKEi01) derivative, respectively. YdfG production was induced in mid-log growing cultures by addition of 30 μg ml^-1^ bacitracin. The cells were harvested 30 min post-induction and disrupted by sonication. For each sample, 10 μg of total protein was separated on a 14% tricine gel and subsequently stained by colloidale Coomassie staining solution. The result is shown in Figure 5. For both derivatives, a clear additional band can be observed in the induced fractions at ~17 kDa. As expected, the YdfG yield received from the pLIKE-rep derivative is much higher compared to the integrative one. To be sure that this band is not a bacitracin effect, control samples of the expression host TMB1151 were treated equally and were also loaded on the gel. Here, no distinct band can be observed in the bacitracin-induced sample (Figure 5). By using the pLIKE-rep derivative, it was possible to achieve a protein yield comparable to the one shown for LiaH in bacitracin-induced *B. subtilis* wild type cells (Figure 1B).

## Conclusions

We have developed a novel and efficient LiaFSR-dependent gene expression system, which places target proteins under the control of an optimized bacitracin-responsive P_*liaI*_ promoter. The LIKE system offers first a single-copy, integrative option (pLIKE-int in strain Bsu-LIKE2), which is genetically stable without selective pressure, but reaches lower protein production levels. The second option consists of the replicative vector pLIKE-rep in combination with strain Bsu-LIKE1 to obtain a maximum gene expression. The LIKE-system has a number of important features: (i) There is no detectable background expression in the non-induced state. (ii) Using bacitracin as inducer, the promoter has an impressive dynamic range of up to 1,000-fold above background level that can be titrated as a function of inducer concentration. (iii) The described range of inducers is wide, including cell wall antibiotics that function as strong (bacitracin, nisin, daptomycin) or intermediately strong (vancomycin) inducers, as well as non-antibiotic conditions that act as intermediate to weak inducers of the Lia-system, including pH-upshift, organic solvents, some detergents, or ethanol [22,25,36-38]. All of these inducers are well-defined and readily available at low prices. Moreover, a recent study demonstrated that the Lia-system can also be induced by the overexpression of certain heterologous and secreted proteins, especially the universal shock protein USP45 from *Lactococcus lactis* and the TEM-1 β-lactamase from *E. coli*[39]. (iv) Lastly, an antibiotic-inducible LIKE-expression strain can easily be converted into a strong constitutive expression platform by the simple deletion of *liaF*, encoding the LiaRS-specific inhibitor protein [21-23]. The effect of such a deletion is shown in Figure 1, which demonstrates that a *liaF* deletion results in a protein production that even surpasses that of the fully induced strains, even in the absence of an inducer.

This flexibility distinguishes the LIKE system from other available expression systems. Taken together, the expression vectors and strains described in this report expand the genetic toolbox already available for protein production, based on the tight and highly dynamic bacitracin-inducible promoter P_*liaI*_. We hope and believe that the vectors and strains described in this report will provide valuable tools for protein expression in *B. subtilis*. The LIKE system, consisting of both expression vectors as well as the host strains Bsu-LIKE1 and Bsu-LIKE2, is available for the scientific community through the *Bacillus* Genetic Stock Center (http://www.bgsc.org; accession numbers ECE255, ECE256 for the two vectors and 1A1070, 1A1071 for the two *B. subtilis* expression strains).

## Methods

### Growth conditions

All bacterial strains (Table 2) were grown in Luria–Bertani (LB) medium at 37°C with aeration. The cell density was determined by measuring the OD_600_ with the Ultrospec™ 2100 pro UV/visible spectrophotometer (GE Healthcare). When appropriate, the growth media were supplemented with chloramphenicol (5 μg mL^-1^), erythromycin (1 μg mL^-1^) plus lincomycin (25 μg mL^-1^) for macrolide-lincosamide-streptogramin (MLS) resistance (*B. subtilis*), or ampicillin (100 μg mL^-1^; *E. coli*). Protein expression was induced by using zinc bacitracin (Sigma).

**Table 2:** Bacterial strains used in this study

**Strain**	**Relevant genotype**	**Source and/or reference**
*E. coli* DH5α	*recA*1 *endA*1 *gyrA*96 *thi hsdR*17(r_K_^-^ m_K_^+^) *relA*1 *supE*44 ϕ80Δ*lacZ*ΔM15 Δ(*lacZYA*-*argF*)*U169*	Laboratory stock
*Bacillus subtilis*		
W168	Wild type, *trpC*2	Laboratory stock
HB0933	W168 *att*SPβ2∆2 *trpC*2, *liaR*::kan	[18]
TMB016	W168 *amy*E::(cat P_*liaI*_-*lacZ*)	[21]
TMB020	HB0933 *amyE*::(cat P_*liaI*_-*lacZ*)	[21]
TMB329	W168 ∆*liaF* (clean deletion)	[23]
TMB331	TMB329 *amyE*::(cat P_*liaI*_-*lacZ*)	This work
TMB408	W168 *amyE*::pSJ5101 (P_*liaI*_-*gfp*)	S. Jordan
TMB604	W168 ΔP_*liaI*_-*liaIH* (clean deletion)	[23]
Bsu-LIKE1 (TMB1151)	W168 ∆*liaIH* (clean deletion)	This work
Bsu-LIKE2 (TMB1152)	W168 ∆*liaIH*-terminator (clean deletion)	This work
TMB1172	W168 *amyE*::pAT6203 (pLIKE-int P_*liaI*(opt)_-*gfp*)	This work
TMB1176	W168 pAT3803 (pLIKE-rep P_*liaI*(opt)_-*gfp*)	This work
TMB1174	TMB604 *amyE*::pAT6203 (pLIKE-int P_*liaI*(opt)_-*gfp*)	This work
TMB1178	TMB604 pAT3803 (pLIKE-rep P_*liaI*(opt)_-*gfp*)	This work
TMB1153	TMB1151 *amyE*::pAT6203 (pLIKE-int P_*liaI*(opt)_-*gfp*)	This work
TMB1342	TMB1151 pAT3803 (pLIKE-rep P_*liaI*(opt)_-*gfp*)	This work
TMB1318	TMB1152 *amyE*::pAT6203 (pLIKE-int P_*liaI*(opt)_-*gfp*)	This work
TMB1343	TMB1152 pAT3803 (pLIKE-rep P_*liaI*(opt)_-*gfp*)	This work
TMB1566	TMB1151 pKSLIKEr01 (pLIKE-rep P_*liaI*(opt)_-His_6_-*ydfG*)	This work
TMB1570	TMB1152 pKSLIKEi01 (pLIKE-int P_*liaI*(opt)_-His_6_-*ydfG*)	This work

### DNA manipulations, transformation and PCR

All plasmid constructions were done in *E. coli* and isolated by alkaline lysis method [40], then used to transform *B. subtilis*[41]. Procedures for DNA manipulation and transformation of *E. coli* were carried out as described [42]. The primers used in this study are listed in Table 3. For all PCR reactions the Phusion DNA Polymerase (Finnzymes) was used according to the manufacturer’s instructions. Sequencing was performed in-house by the Sequencing Facility of the LMU Biocenter.

**Table 3:** Oligonucleotides used in this study

**Primers**	**Sequence (5' to 3')**^**a**^	**Description/position**
Plasmid construction		
TM2064	CAT*GGTCTC*AGATCTTTAAAACGCCATGCCTCG	BsaI; 5' end of P_*liaI*_
TM1980	CTTGTT*GGATCCATCGAT*GAT**CCTCCT**TACGTTTTCCTTGTCTTC	Strong SD region; BamHI, ClaI; 3' end of P_*liaI*_
TM1991	ATCT*GAATTC*GGTTTTAAAACGCCATGCC	EcoRI; 5' end of P_*liaI*_
TM1992	ATTTTC*TCTAGA*AT**CCTCCT**TACGTTTTCCTTGTCTTC	Strong SD region; XbaI; 3' end of P_*liaI*_
TM1981	TCCT*ATCG****ATG***AGTAAAGGAGAAGAACTTTTCACTGG	ATG start codon; ClaI; 5' end of *gfpmut1*
TM1982	GGCC*AAGCTT*GAACTAGTTTCATTTATTTGTAGAGC	HindIII; 3' end of *gfpmut1*
TM1993	TTCC*TCTAG****ATG***AGTAAAGGAGAAGAACTTTTC	ATG start codon; XbaI; 5' end of *gfpmut1*
TM1994	GGCC*GTCGAC*GAACTAGTTTCATTTATTTG	SalI; 3' end of *gfpmut1*
TM2535	CCAT*ATCG****ATG****CATCATCATCATCATCAC*GAAACGAGATTTCTAATGGAAAAAG	ATG start codon; ClaI; His_6_-tag; 5’ end of *ydfG*
TM2536	CCAT*AAGCTT*TCAATCTGCTGCGGGCATTTTC	HindIII; 3’ end of *ydfG*
TM2545	CCAT*TCTAG****ATG****CATCATCATCATCATCAC*GAAACGAGATTTCTAATGGAAAAAG	ATG start codon; XbaI; His_6_-tag; 5’ end of *ydfG*
Clean deletions		
TM2130	GCGG*GGATCC*TCTTACATTTATTAGTCC	BamHI; upstream of P_*liaI*_
TM2131	CATTTGCCGCTTTTGTCTGGGCAGATCCTCCTTTCGTTTTC	3' end of P_*liaI*_; 3' end of *liaH*
TM1055	CCAGACAAAAGCGGCAAATG	3' end of *liaH*
TM1058	CCAT*GAATTC*GAATGCGGACGTCCGTCACGC	EcoRI; inside the *liaG* gene
TM2132	GCGAATTGATACGTGCGGGCAGATCCTCCTTTCGTTTTC	upstream of *liaI* gene; upstream of *liaG*
TM2133	CCGCACGTATCAATTCGC	upstream of *liaG*
TM2134	GCTA*GAATTC*TGCCGGCTGTTTTGGAG	EcoRI; center of *liaG* gene

^a^ Relevant restriction sites are shown in italics, complementary regions for joining PCR are underlined. The sequence for optimized SD sequences of the *liaI* promoter is indicated by bold type, the start codon is bold italic. The His-tags are in italics and underlined.

### Construction of markerless deletion mutant strains

Several markerless deletions of the *liaIH* operon (including its promoter and terminator) were constructed using the vector pMAD [43]. Genomic regions of approximately 1 kb up- and downstream of the regions to be deleted were amplified using the primers listed in Table 3. The two fragments were fused in a second joining PCR reaction, and the resulting fragment was cloned into pMAD via BamHI and EcoRI, generating the plasmids pAT101 (Δ*liaIH*) and pAT102 (Δ*liaIH*_Terminator_). For generating the deletion mutants, the procedure described by Arnaud *et al.* was applied [43]. In brief, *B. subtilis* 168 was transformed with pAT101 or pAT102 (Table 4) and incubated for two days at 30°C on LB agar plates containing X-Gal (5-bromo-4-chloro-3-indolyl-β-D-galactopyranoside; 100 μg mL^-1^) with MLS selection. Individual blue colonies were selected and incubated for 6 to 8 h at 42°C in LB medium with MLS selection, resulting in the integration of the plasmids into the chromosome. Blue colonies were again picked from LB (X-Gal) plates and incubated at 30°C for 6 h in LB medium without selection. Subsequently, the liquid culture was shifted to 42°C for 3 h, and the cells were then plated on LB (X-Gal) plates, this time without selective pressure. White colonies that had lost the plasmids were picked and checked for MLS sensitivity. Finally, strains TMB1151 (Δ*liaIH*) and TMB1152 (Δ*liaIH*_Terminator_) were analyzed by PCR and sequencing to confirm the integrity of the desired genetic modifications.

**Table 4:** Vectors and plasmids used in this study

**Plasmid**	**Genotype/properties**^**a**^	**Primer pair(s) used for cloning**	**Reference**
pDG1662	*cat*, *spc*, *bla*, *amyE'* … *'amyE* integrative vector		[28]
pGP380	*erm*, *bla*, Strep-Tag, PdegQ36, replicative vector		[29]
pMAD	*erm*, ori(pE194-Ts), MCS-P_*clpB*_-*bgaB*, *ori*(pBR322), *bla*		[43]
pSG1151	*bla*, *cat*, *gfpmut1*		[44]
pAT6200	pDG1662 derivative; *spc* gene deleted		This work
pLIKE-int	pAT6200 derivative; P_*liaI*(opt)_; integrative protein expression vector	TM2064/TM1980	This work
pLIKE-rep	pGP380 derivative; P_*liaI*(opt)_; replicative protein expression vector	TM1991/TM1992	This work
pAT6203	pLIKE-int, P_*liaI*(opt)_ translationally fused to *gfp*	TM1981/TM1982	This work
pAT3803	pLIKE-rep, P_*liaI*(opt)_ translationally fused to *gfp*	TM1993/TM1994	This work
pAT101	pMAD Δ*liaIH* up/down overlap	TM2130/ TM2131, TM1055/ TM1058	This work
pAT102	pMAD Δ*liaIH*_Terminator_ up/down overlap	TM2130/ TM2132, TM2133/ TM2134	This work
pKSLIKEr01	pLIKE-rep, P_*liaI*(opt)_ translationally fused to His_6_-*ydfG*	TM2545/TM2536	This work
pKSLIKEi01	pLIKE-int, P_*liaI*(opt)_ translationally fused to His_6_-*ydfG*	TM2535/TM2536	This work

^a^ Resistance cassettes: *erm*, erythromycin; *bla*, ampicillin; *cat*, chloramphenicol; *spc*, spectinomycin.

### Plasmid and strain construction

Bacterial strains used in this study are derivates of the laboratory wild type strain *B. subtilis* W168 and are listed in Table 2. Plasmids used in this study are listed in Table 4. The promoter of the *liaIH* operon for integrative and replicative vectors was obtained from strain *B. subtilis* W168 by PCR, using primers TM2064/TM1980 and TM1991/TM1992 (Table 3), respectively. During the amplification, bases in the ribosome-binding site (RBS) were mutated to a strong *B. subtilis* Shine-Dalgarno (SD) sequence (TAAGGAGG) [27] to yield the optimized *liaI* promoter P_*liaI*(opt)_.

The integrative expression vector pLIKE-int, containing P_*liaI*_ with an optimized SD site (P_*liaI*(opt)_) was generated in two steps. First, the *B. subtilis* integrative vector pDG1662 was treated with BstBI to remove the spectinomycin resistance gene. The truncated (6141 bp) fragment was self-ligated, yielding vector pAT6200. During this step, the multiple cloning site (MCS), containing unique BamHI, HindIII, and EcoRI sites was expanded by an additional unique ClaI restriction site, which is required for introducing genes at the ATG start codon (see Figure 2 for details): a PCR product encompassing P_*liaI*(opt)_ was digested with BsaI and BamHI and cloned into pAT6200 digested with BamHI, resulting in pLIKE-int. The ClaI restriction site is recommended to use for reconstruction of the ATG start codon, but it is not strictly necessary. The use of BamHI, HindIII, or EcoRI has the disadvantage of fusing additional amino acids to the N-terminus of the target protein which can cause undesired disabilities.

To construct the replicative expression vector pLIKE-rep, again harboring P_*liaI*(opt)_, the promoter fragment was amplified by PCR using primers TM1991/TM1992 (Table 3). After digest of the PCR product with EcoRI and XbaI, the promoter region was ligated into the corresponding sites of pGP380, resulting in vector pLIKE-rep. For cloning of a gene into pLIKE-rep, XbaI must be used as restriction enzyme to generate the ATG start codon (see Figure 2).

For the determination of the properties of the two expression vectors, the genes *gfpmut1* and *ydfG* were used. The *gfpmut1* gene was amplified using primers TM1981/TM1982 and TM1993/TM1994, respectively (Table 3), using plasmid pSG1151 as the template. The 720-bp amplicon obtained was cloned into ClaI/HindIII-digested pLIKE-int or XbaI/SalI-digested pLIKE-rep, resulting in translational fusions with P_*liaI*(opt)_ in pAT6203 and pAT3803, respectively (Table 4). Next, the *B. subtilis* strains W168, TMB604, TMB1151, and TMB1152 (Table 2) were transformed with the pAT6203 integrative plasmid. The resulting strains were designated TMB1172, TMB1174, TMB1153, TMB1318, respectively (Table 2). Strains bearing the replicative pAT3803 GFP-expression plasmid were constructed by transformation of the above strains with plasmid DNA and selection for MLS resistance, resulting in strains TMB1176, TMB1178, TMB1342, and TMB1343, respectively. The *ydfG* gene was amplified from *Bacillus licheniformis* genomic DNA using primers TM2545/TM2536 and TM2535/TM2536, respectively (Table 3). The PCR product was cloned into ClaI/HindIII digested pLIKE-int or XbaI/HindIII digested pLIKE-rep, resulting in plasmids pKSLIKEi01 and pKSLIKEr01, respectively (Table 4). Next, the *B. subtilis* strain TMB1151 was transformed with pKSLIKEr01 replicative plasmid and TMB1152 was transformed with the linearized pKSLIKEi01 integrative plasmid, resulting in strains TMB1566 and TMB1570 (Table 2).

### Activation of P_*liaI*_ by bacitracin and analysis of *gfp* gene expression

For bacitracin-mediated induction of gene expression, the appropriate *B. subtilis* strains were inoculated from overnight LB cultures into a final volume of 150 μL LB medium in a 96-well plate with optical bottom (Sarstedt) and were incubated in a Synergy™ 2 multimode microplate reader (Biotek) at 37°C with constant medium shaking. When the culture reached an OD_600_ of 0.45, bacitracin (30 μg mL^-1^ final concentration) was added to one half of the wells (induced sample), and the other half was left untreated (uninduced control). Plates were covered with lids to prevent evaporation and incubated for 4 h. Growth was monitored by measuring absorbance at 600 nm. Fluorescence readings were taken from the bottom by using a GFP-specific filter pair (excitation 485/20 nm, emission 528/20 nm). Measurements were taken in 10 min intervals. To calculate expression levels, the natural fluorescence of three cultures of wild type *B. subtilis* strain 168 (containing no reporter gene) were averaged and subtracted from the raw fluorescence value of each reporter strain at the same OD_600_ value [45]. Determination of P_*liaI*_ activity was calculated as described in [45] as the derivative of the fluorescence divided by the OD_600_ (dGFP/dt/OD_600_) for each time point. Expression values were averaged from three independent samples of the same time points ((P1+P2+P3)/3). Polynomial and exponential functions were used to fit the experimental GFP dataset; promoter activities (dGFP/dt/OD_600_) were calculated using these functions [45].

### Western blotting

Total cytoplasmic proteins were prepared from 15 mL culture per time point by sonication. Proteins (20 μg per lane) were separated by SDS-PAGE, according to standard procedure [42]. After electrophoresis and equilibration of the gels in transfer buffer [15.2 g Tris; 72.1 g glycine; 750 mL methanol (100%) in a final volume of 5 L with deionized water] the proteins were blotted to a PVDF membrane using a mini-trans blot apparatus (Bio-Rad) according to standard procedure [42]. The LiaH antibody (polyclonal rabbit antisera raised against purified His10–LiaH [46]), GFP antibody (rabbit monoclonal antibody against the green fluorescent protein, Epitomics), and the secondary antibody (anti-rabbit IgG HRP conjugate, Promega) were diluted 1:20,000, 1:3,000, and 1:100,000, respectively. For LiaH/GFP detection, AceGlow^TM^ (PeqLab) was used according to the manufacturer’s instructions. Blots were documented on a QUANTUM-ST4-3026 chemiluminescence documentation system (PeqLab).

### Overproduction of YdfG

For the overexpression of *ydfG*, strains TMB1566 and TMB1570 were grown in LB medium at 37°C until they reached an OD_600_ of ~0.4-0.5. Cultures were split and one half was induced with 30 μg ml^-1^ bacitracin for 30 min. The other half was left untreated. 20 ml of each culture was harvested by centrifugation and cell pellets were kept at −80°C until further use. For total protein preparation, the cell pellets were resuspended in 1 ml of cold disruption buffer (50 mM Tris–HCl, 100 mM NaCl, pH 7.5) and cells were disrupted by sonication on ice. Proteins (10 μg per lane) were separated by 14% tricine SDS-PAGE, according to standard procedure [47] and gels were subsequently stained by colloidale Coomassie staining solution [48].

## Competing interests

The authors declare that they have no competing interests.

## Authors’ contributions

AAT carried out all experiments with the exception of the overexpression experiment and those acknowledged below. KS performed the overexpression experiment with YdfG. AAT, KS, and TM conceived the study and wrote the manuscript. MRS participated in its design and coordination and helped to draft the manuscript. All authors read and approved the final manuscript.
